# Myosin Light Chain Kinase Modulates to Improve Myocardial Hypoxia/Reoxygenation Injury

**DOI:** 10.1155/2022/8124343

**Published:** 2022-03-26

**Authors:** Qibo Zhang, Xiaoxiao Liu, Wen Yi, Chunquan Zhang

**Affiliations:** ^1^Department of Ultrasound, Weihai Municipal Hospital, Weihai, Shandong 264200, China; ^2^Department of Cardiology, People's Hospital of Gaotang County, Liaocheng, Shandong 252800, China; ^3^Department of Cardiology, People's Hospital of Huantai County, Zibo, Shandong 256400, China; ^4^Department of Cardiology, Qingdao Municipal Hospital, Qingdao, Shandong 266000, China

## Abstract

**Objective:**

The aim of this study was to evaluate whether myosin light chain kinase (MLCK) knockdown attenuated H9C2 cell hypoxia/reoxygenation (H/R) injury and downstream signaling pathway.

**Methods:**

The MLCK expression in H/R injury model H9C2 cell was determined by western blot and qRT-PCR. H/R cells were transfected with si-MLCK in the presence of P38 inhibitor (SB203580) or ERK inhibitor (U0126). Then, cell apoptosis was verified by flow cytometry. Apoptosis-related proteins were detected by western blot. The contents of reactive oxygen species (ROS), lactate dehydrogenase (LDH), superoxide dismutase (SOD), interleukin-6 (IL-6), interleukin (IL)-1*β* (IL-1*β*), and tumor necrosis factor-*α* (TNF-*α*) were measured using flow cytometry and colorimetric assays, respectively.

**Results:**

MLCK expression was higher in H/R cells. Knockdown of MLCK diminished the amounts of ROS, LDH, IL-6, IL-1*β,* and TNF-*α* and elevated the release of SOD in H/R model H9C2 cells. Additionally, H/R injury induced the cumulative expression and phosphorylation of ERK and the phosphorylation of P38, whereas MLCK siRNA-treated cells showed decreased ERK1/2 and P38 activation. Inversely, P38 inhibitor (SB203580) and ERK inhibitor (U0126) could reverse the cardioprotective effects induced by si-MLCK.

**Conclusion:**

MLCK knockdown attenuated H/R injury in H9C2 cells via regulating the ERK/P38 signaling pathway. MLCK/ERK/p38 axis may provide novel insight into therapeutic targets to restrain I/R injury caused by revascularization therapy after acute myocardial infarction.

## 1. Introduction

Restoration of blood flow to ischemic myocardium is considered one of the most effective treatment strategies for acute myocardial infarction [1]. Restoring blood supply minimizes damage caused by the acute myocardial infarction, thus decreasing the rate of mortality [2]. However, revascularization may aggravate cardiovascular trauma and produce a second blow to the myocardium, referred to as myocardial ischemia/reperfusion (I/R) injury, which can induce serious consequences, such as myocyte necrosis and apoptosis and cardiac arrest [3]. Proper and timely restoration of blood flow through antithrombotic drugs or mechanical intervention is the main treatment [4]. However, these treatments are limited by narrow time windows and side effects. In addition, reperfusion can cause secondary injury. Many mechanisms have been proposed to mediate reperfusion induced injury, including inflammation and metabolic changes [5]. Exploring prevention strategies and action mechanisms of I/R injury to reduce the damage following I/R has vital research significance for improving the overall survival and prognosis of patients with cardiovascular disease.

Myosin light chain kinase (MLCK) is encoded by the MYLK3 gene. Cardiac MLCK phosphorylates of cardiac myosin regulatory light chain (cMLC2) play critical role in regulating the rate and the force of contraction and barrier function of the endothelium [6]. Sarah et al. found that MLCK inhibitor ML-7 effectively impeded cytokine-induced endothelial barrier dysfunction [7]. Huang et al. showed that the elevated MLC2 phosphorylation by MLCK suppressed cardiac hypertrophy by contributing to potentiate contractile performance and efficiency [8] whereas few research studies are available to elucidate how MLCK influences the biological behaviors of I/R.

Mitogen-activated protein kinase (MAPK) is a serine/threonine specific protein kinase. By introducing extracellular stimuli into cells, MAPK plays an important role in cell biological activities, including proliferation, differentiation, and apoptosis. MAPKs include 3 families: cJun N-terminal kinases (JNKs), extracellular signal-regulated kinases (ERKs), and p38 MAPK. It is reported that inhibition of cardiac p38 MAPK pathway can delay ischemic cell death and protect myocardial mitochondria from I/R injury [9]. Moreover, the mitogen-activated protein kinase (MAPK) signaling pathway exerts a crucial role in cardiovascular disease [10, 11]. However, the relationship between MAPK and MLCK in I/R injury has not been clarified. In this study, hypoxia/reoxygenation (H/R) is utilized as an in vivo model of myocardial I/R injury to explore the function of the MLCK/ERK/P38 axis in I/R injury.

## 2. Materials and Methods

### 2.1. Cell Cultures and H/R Treatment

Rat embryonic-heart-derived cell line H9C2 cells were purchased from Wuhan University. To establish a cellular H/R model, the trypsin was used to digest the cells, and then the cells were maintained in serum-free DMEM and maintained in anaerobic conditions. After that, the cells were resuspended with normal medium to the normoxic incubator at 37°C under 5% CO_2_ incubation for 4 h to undergo reoxygenation. Then, H/R cells were transfected with si-MLCK in the presence of 20 *μ*mol/L P38 inhibitor (SB203580) for 1 h or 10 *μ*M ERK inhibitor (U0126) for 30 min and then utilized in subsequent experiments.

### 2.2. Transfection

To generate inducible MLCK-low-expression cells, H9C2 cells were plated on a 100 mm dish (Corning). At 50–70% confluence, cells were then transfected with 25 nM MLCK siRNA or siRNA for 48 h. These plasmids were obtained from Thermo Scientific, Rockford, IL, USA. Transfection was performed according to Lipofectamine RNAiMAX reagent protocol (Invitrogen).

### 2.3. Quantitative Real-Time PCR

Total RNA was extracted from H9C2 cells by TRIzol. cDNA was synthesized using the SuperScript II (Invitrogen). The mRNA levels of MLCK were quantified using the SYBR mixture (Takara Biotechnology co., ltd.) on an ABI-7900 System. GAPDH levels were utilized as references for MLCK. All mRNA expression was calculated by 2^−ΔΔct^. The primers were synthesized by GenePharma. MLCK forward: 5′-GCTGCCTGACCACGAATATAAG-3′, reverse: 5′-GACACCATCCACTTCATCCTTC-3′. GAPDH forward: 5′-CGCTAACATCAAATGGGGTG-3′ and reverse: 5′-TTGCTGACAATCTTGAGGGAG-3′.

### 2.4. Measurement of IL-6, IL-1*β*, TNF-*α*, LDH, SOD, and ROS Production

The levels of IL-6, IL-1*β*, TNF-*α*, LDH, and SOD were detected by ELISA assay kits. Then, the intensity was measured by a microplate reader to make a visualization of color intensity development. DCFH-DA probe was used to measure ROS production in H9C2 cells. H9C2 cells were incubated in DCFH-DA and then washed with serum-free DMEM three times. Then, the fluorescence intensity was detected under a fluorescence microscope (Olympus Corporation). The wavelength of stimulated light was 485 nm, and the wavelength of emission light was 525 nm.

### 2.5. Evaluation of Apoptosis

After 48 h incubation, the cells were washed three times with cold PBS, harvested, and resuspended with annexin-binding buffer and then incubated with Annexin V-FITC and PI solution. Cellular fluorescence was detected by flow cytometer.

### 2.6. Western Blot Assay

H9C2 cells were exposed to ML-7 for 48 h in the presence of 20 *μ*mol/L SB203580 for 1 h or 10 *μ*M U0126 for 30 min. Protein concentration was quantified by BCA kit. Later, the protein samples were separated on SDS-PAGE and transferred onto PVDF membranes. The PVDF membranes were blocked with 5% skim milk, then incubated with primary antibodies, such as p-P38, P38, ERK, p-ERK, Bax, Bcl-2, GAPDH, and MLCK, and then incubated with the HRP-conjugated secondary antibodies. Finally, the blots were visualized by ECL reagent.

### 2.7. Statistical Analysis

Result values were expressed as means ± SD. Statistical significance of multiple groups was assessed by one-way ANOVA. Pairwise comparisons were analyzed by Student's *t*-test. *p* < 0.05 was considered statistically significant.

## 3. Results

### 3.1. MLCK Expression Was Upregulated in H/R Cells

**Figure 1:**
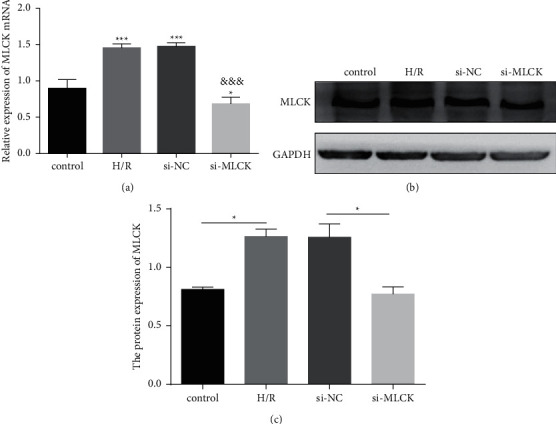
The MLCK expression was up-regulated in H/R cells. (a) The MLCK mRNA expression in H/R cells was measured by qRT-PCR assay. (b-c) The MLCK expression in H/R cells was measured by western blot vs. control, ^*∗*^*p* < 0.05, ^∗∗^*p* < 0.01, ^∗∗∗^*p* < 0.001 vs. H/R; &*p* < 0.05, &&&*p* < 0.001.

To determine the association between MLCK expression and heart ischemia-reperfusion injury, H9C2 cells were used as a representative in vitro model of heart ischemia-reperfusion injury. First, siRNA against MLCK was performed to suppress MLCK expression in H9C2 cells. Then, we performed qRT-PCR and western blot analysis in heart ischemia-reperfusion injury cell model. MLCK mRNA expression was increased in H/R cells, whereas the si-MLCK obviously hindered the increase induced by hypoxia/reoxygenation injury (Figure 1(a)). Additionally, the result of western blot showed that MLCK protein expression was elevated in H/R cells, while si-MLCK remarkably hampered the accumulation-mediated by H/R injury (Figure 1(b)). We, therefore, hypothesized that MLCK might relate to the hypoxia/reoxygenation injury.

### 3.2. MLCK Knockdown Reduces Hypoxia/Reoxygenation Injury

**Figure 2:**
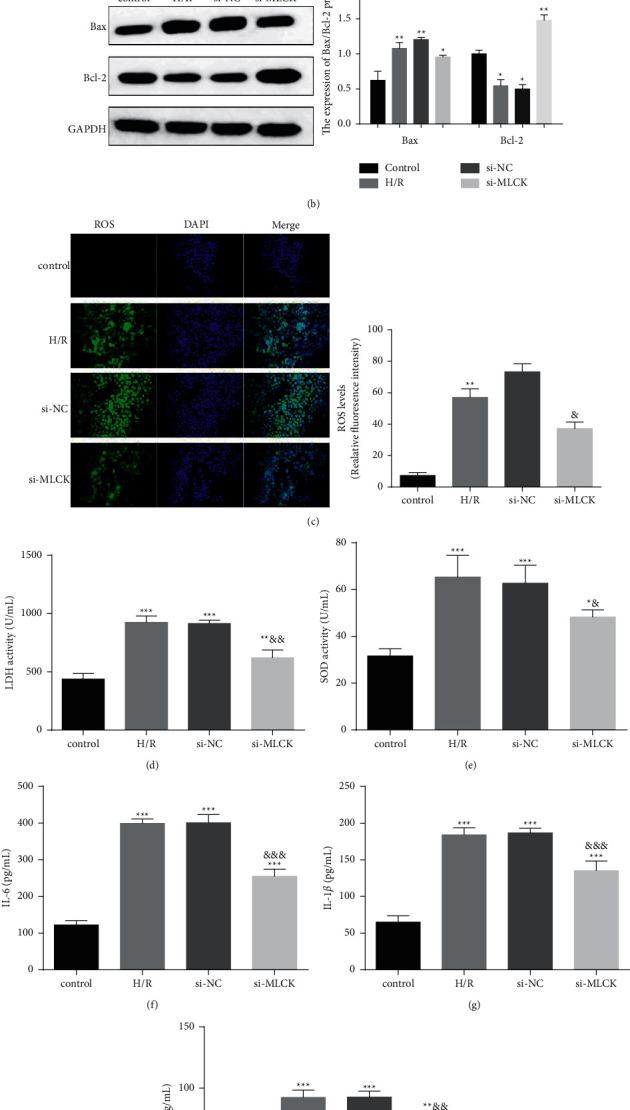
MLCK knockdown reduces hypoxia/reoxygenation injury. (a) The apoptotic rate in H/R cells was determined by flow cytometry. (b) The Bax and Bcl-2 protein expression in H/R cells was detected by western blot. (c) DCFH-DA probe was used to measure ROS production in H9C2 cells. (d–h) The production of ROS, LDH, and SOD in H/R cells were detected by ELISA assay kits vs. control, ^*∗*^*p* < 0.05, ^∗∗^*p* < 0.01, ^∗∗∗^*p* < 0.001. Vs. H/R; ^&^*p* < 0.05, ^&&^*p* < 0.01, ^&&&^*p* < 0.001.

The results showed that the H/R injury markedly increased the apoptotic rate (20.72 ± 0.46%) compared with the control group (6.72 ± 0.87%), whereas the apoptotic rate was significantly decreased in MLCK knockdown H/R cells (9.04 ± 1.03) (Figure 2(a)). Bcl-2 family members play a vital role in releasing mitochondrial apoptosis factors, so we detected the Bax and Bcl-2 expression (Figure 2(b)). H/R injury facilitated suppression in Bcl-2 expression and augmentation in Bax expression. Inversely, si-MLCK could compromise the phenomena induced by heart ischemia-reperfusion injury. In addition, H/R injury elicited augmentation in production of ROS, LDH, IL-6, IL-1*β,* and TNF-*α* and a decrease in the release of SOD. In contrast, depletion of MLCK suppressed the levels of oxidative damage marker (ROS and LDH) and inflammatory cytokines and increased the oxygen free radical scavenger (SOD) (Figures 2(c)–2(h)). The results indicate that MLCK knockdown can block H/R injury in vitro.

### 3.3. MLCK Knockdown Reduces Hypoxia/Reoxygenation Injury via ERK/P38 Pathway

**Figure 3:**
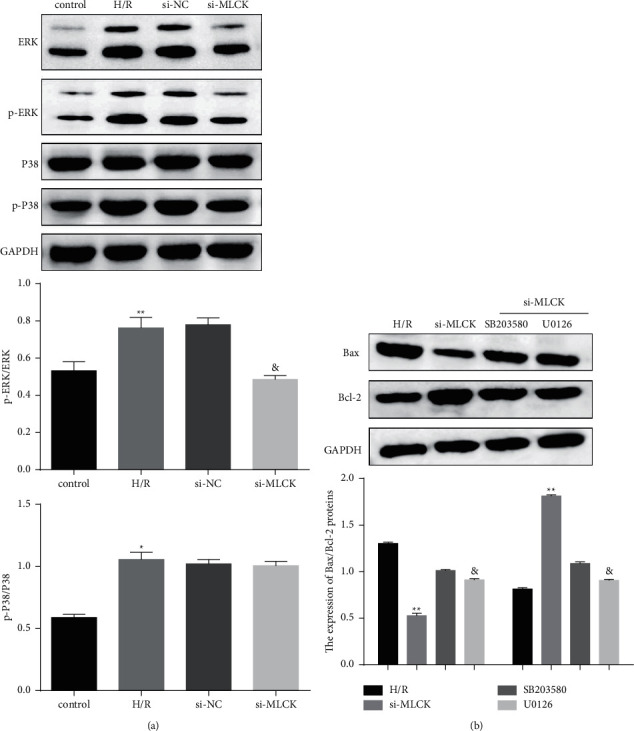
MLCK knockdown regulated ERK/P38 pathway and Bax/Bcl-2 expression. (a) The expression of ERK and P38 and phosphorylation in H/R cells was detected by western blot. (b) The Bax and Bcl-2 expression in H/R cells after ERK/P38 inhibitors treatment was detected by western blot vs. control, ^*∗*^*p* < 0.052, ^∗∗^*p* < 0.01, ^∗∗∗^*p* < 0.001. vs. H/R, ^&^*p* < 0.05.

**Figure 4:**
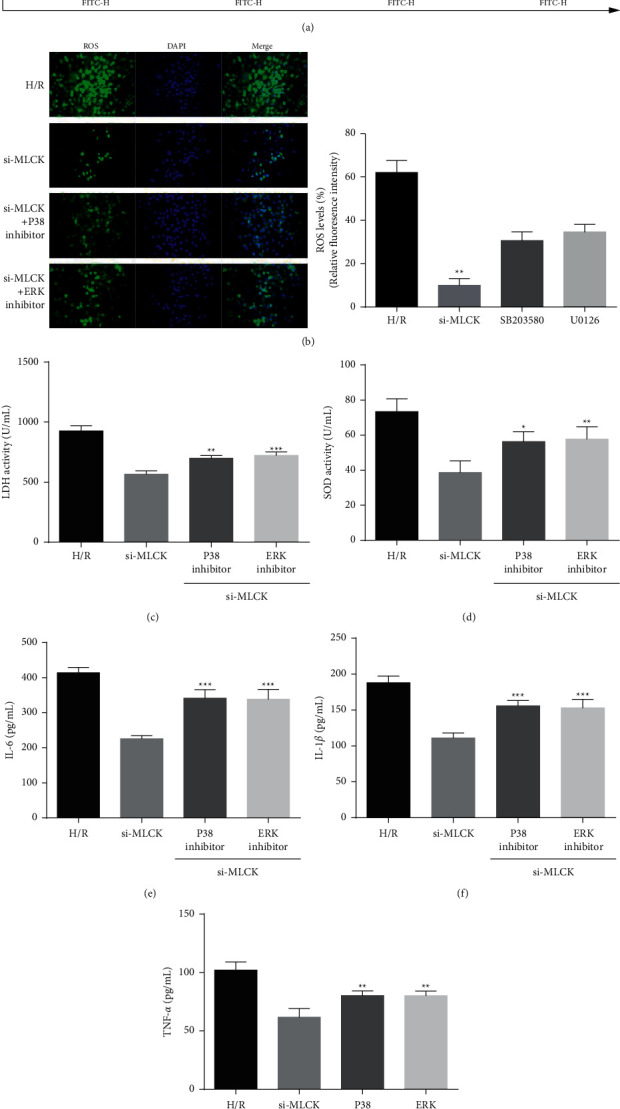
MLCK knockdown reduces hypoxia/reoxygenation injury via ERK/P38 pathway. (a) The apoptotic rate in H/R cells after ERK/P38 inhibitors (U0126/SB203580) treatment was determined by flow cytometry. (b) DCFH-DA probe was used to measure ROS production in H9C2 cells. (c–g) The production of LDH, SOD, IL-6, IL-1*β*, and TNF‐*α* after ERK/p38 inhibitors treatment were detected by ELISA assay kits. (e–g) The inflammatory cytokines after ERK/p38 inhibitors treatment were detected by ELISA assay kits vs. H/R; ^*∗*^*p* < 0.052, ^∗∗^*p* < 0.01, ^∗∗∗^*p* < 0.001.

H/R injury is affected by molecular changes that involve the activity of multifarious signaling networks, including ERK and P38 signaling [12]. In the current study, we hypothesized that depletion of MLCK affects ERK and P38 signaling pathways and thereby mediates apoptosis, oxidative damage, and inflammatory cytokines release. H/R injury induced the cumulative expression and phosphorylation of ERK1/2 and the phosphorylation of P38, whereas MLCK siRNA-treated cells showed decreased ERK1/2 and P38 activation (Figure 3(a)). Additionally, the suppression of Bax expression and augmentation of Bcl-2 expression facilitated by MLCK knockdown were reversed by U0126 and SB203580, respectively (Figure 3(b)). To explore the correlation between H/R injury induced by MLCK and ERK/P38 signaling pathway, ERK/P38 inhibitors were used. Interestingly, the inhibition of ERK activation with the U0126 and the inhibition of P38 activation with the SB203580 decreased apoptosis induced by si-MLCK, respectively, which facilitated the augmentation of apoptosis rate (Figure 4(a)). In addition, we further investigated the effect of ERK/P38 signaling pathway on oxidative damage and inflammatory cytokines release of H9C2 cells. These results demonstrated that the suppression of oxidative damage marker (ROS and LDH) and the augmentation of oxygen free radical scavenger (SOD) induced by MLCK knockdown were compromised by U0126 and SB203580, respectively (Figures 4(b)–4(d)). Moreover, the attenuation of inflammatory cytokines including IL-6, IL-1*β*, and TNF-*α* induced by MLCK knockdown was reversed by U0126 and SB203580, respectively (Figures 4(e)–4(g)). Collectively, these data suggest that MLCK knockdown reduces H/R injury via ERK/P38 pathway.

## 4. Discussion

I/R injury reduces the activity of vascular endothelial cells and cardiomyocytes, vitiates cardiac function, aggrandizes the area of myocardial infarction, and increases the patient's risk of death [13, 14]. The present study substantiates ERK/P38 co-activation as the decisive signaling mechanism by which MLCK rescues myocardium from IR-induced inflammation, oxidative stress, and apoptosis. Our findings were further confirmed by administering the ERK inhibitor (U0126) and P38 inhibitor (SB203580), which revoked MLCK knockdown mediated cardioprotective effect via suppressing ERK/P38 phosphorylation.

The mechanism of IR is a complicated procedure, which is intimately associated with the oxygen free radical system. The injured endothelial and myocardial cells generate oxygen free radicals, which exacerbates cell membrane damage and lead to cardiac dysfunction or injury [15]. Additionally, the inflammatory response incites continuously release such as IL-6, TNF-*α*, and IL-1*β* [16]. Moreover, it has been well established that myocardial apoptosis contributes to I/R injury [17]; thereby, the inhibition of inflammation, oxygen free radicals and apoptosis could minimize cardiac damage-induced I/R and therefore alleviate or even restrain the occurrence of heart failure.

MLCK participated in the pathology of various cardiovascular disorders, for instance, heart failure, cardiac hypertrophy, and myocardial infarction. Previously, studies have shown that the elevated MLC2 phosphorylation attenuates cardiac hypertrophy by potentiating contractile efficiency and performance [8]. Shan Hu et al. demonstrated that the miR-200c knockdown provides considerable protective effects against AngII-induced cardiac hypertrophy in cardiomyocytes by targeting MLCK and blocking ROS production and apoptosis [18]. Moreover, Massengill MT et al. showed that the down-regulation of cMLCKin *Mylk3*-KO mice was correlated with heart failure [19]. In this evaluation, we found that MLCK was elevated in I/R injury cells, and the knockdown of MLCK could compromise I/R injury-induced apoptosis, pro-inflammatory cytokine release (IL-6, TNF-*α*, and IL-1*β*), ROS, and oxygen free radical scavenger (LDH and SOD). We provided in vitro experimental evidence that supports the concept that MLCK is a decisive regulator for I/R injury.

P38 mitogen-activated protein kinase (MAPK) plays a crucial role in myocardial apoptosis and ventricular remodeling. Suppressing the p38 MAPK signaling pathway via specific inhibitors can alleviate myocardial apoptosis and ventricular remodeling [20]. For instance, ZHOU et al. found that isoflurane exerts a protective effect against the cardiac function of rats from myocardial I/R injury, decreases the myocardial infarction area, alleviates the oxidative stress response, and reduces the pathological damage in myocardial cells via blocking the p38 MAPK signaling pathway [21].

An extracellular signal-regulated kinase (ERK) is the vital member of the MAPK family, which can modulate cell proliferation and differentiation [22]. The activated ERK signaling pathway promotes the expression of hypoxia-inducible factor 1 (HIF-1) under the state of myocardial ischemia, eventually adapts to hypoxia. Current studies have demonstrated that the ERK1/2 signaling pathway exerts a cardioprotective role in myocardial I/R injury. Moreover, suppressing the ERK1/2 signaling pathway elevates I/R-induced myocardial apoptosis [10]. In this study, we verified that only 2 subunits of the MAPK family could be activated by H/R stimulation, and ERK and p38 were blocked by the MLCK deficiency. Furthermore, the cardioprotective role induced by MLCK depletion could be reversed by ERK and p38 specific inhibitors via accelerating apoptosis, exacerbating the production of ROS, LDH, IL-6, TNF-*α*, and IL-1*β* and vitiating the release of SOD, respectively.

In conclusion, our research confirmed that MLCK deficiency exerts a protective effect against oxidative stress reaction, inflammatory response, and apoptosis after I/R treatment. The modulating effect of MLCK is generally relying on the regulation of ERK/p38 signaling. Based on these pieces of evidence, MLCK/ERK/p38 axis may provide novel insight into therapeutic targets to restrain I/R injury caused by revascularization therapy after acute myocardial infarction.

## Data Availability

The data used to support the findings of this study are available from the corresponding author upon request.
